# Use of Complementary Therapies Among Primary Care Clinic Patients With Arthritis

**Published:** 2004-09-15

**Authors:** Carla J. Herman, Peg Allen, Arti Prasad, William C. Hunt, Teresa J. Brady

**Affiliations:** Department of Internal Medicine, School of Medicine, University of New Mexico Health Sciences Center; Department of Internal Medicine, University of New Mexico Health Sciences Center, Albuquerque, NM; Department of Internal Medicine, University of New Mexico Health Sciences Center, Albuquerque, NM; Epidemiology and Cancer Control, University of New Mexico Health Sciences Center, Albuquerque, NM; Arthritis Program, Division of Adult and Community Health, National Center for Chronic Disease Prevention and Health Promotion, Centers for Disease Control and Prevention, Atlanta, Ga

## Abstract

**Introduction:**

Use of complementary and alternative medicine (CAM) for chronic conditions has increased in recent years. There is little information, however, on CAM use among adults with clinic-confirmed diagnoses, including arthritis, who are treated by primary care physicians.

**Methods:**

To assess the frequency and types of CAM therapy used by Hispanic and non-Hispanic white women and men with osteoarthritis, rheumatoid arthritis, or fibromyalgia, we used stratified random selection to identify 612 participants aged 18–84 years and seen in university-based primary care clinics. Respondents completed an interviewer-administered survey in English or Spanish.

**Results:**

Nearly half (44.6%) of the study population was of Hispanic ethnicity, 71.4% were women, and 65.0% had annual incomes of less than $25,000. Most (90.2%) had ever used CAM for arthritis, and 69.2% were using CAM at the time of the interview. Current use was highest for oral supplements (mainly glucosamine and chondroitin) (34.1%), mind-body therapies (29.0%), and herbal topical ointments (25.1%). Fewer participants made current use of vitamins and minerals (16.6%), herbs taken orally (13.6%), a CAM therapist (12.7%), CAM movement therapies (10.6%), special diets (10.1%), or copper jewelry or magnets (9.2%). Those with fibromyalgia currently used an average of 3.9 CAM therapies versus 2.4 for those with rheumatoid arthritis and 2.1 for those with osteoarthritis. Current CAM use was significantly associated with being female, being under 55 years of age, and having some college education.

**Conclusion:**

Hispanic and non-Hispanic white arthritis patients used CAM to supplement conventional treatments. Health care providers should be aware of the high use of CAM and incorporate questions about its use into routine assessments and treatment planning.

## Introduction

Rheumatic conditions such as arthritis and chronic joint pain are the leading cause of limitation in daily activities and disability among adults in the United States (1). In 2001, self-reported data obtained by the U.S. Behavioral Risk Factor Surveillance System indicated that a third of American adults had chronic joint symptoms or physician-diagnosed arthritis (1). Researchers estimate that by 2030, some 41 million adults aged 65 and older will have arthritis or chronic joint symptoms (2). Management of chronic rheumatic conditions usually consists of physician-prescribed medications, physician-recommended over-the-counter medications, physical therapy, and self-management strategies recommended by physicians and the American College of Rheumatology (3) such as exercise, weight control, use of heat or cold, intermittent rest, and stress management. Increasingly, adults are also adopting complementary and alternative medicine (CAM) to help manage their chronic conditions (4,5).

This study addresses three gaps in the literature on CAM use for arthritis. First, CAM use among adults with physician-confirmed arthritis who are treated by primary care physicians has not been well studied. Previous studies on prevalence of CAM use for arthritis in the United States have typically included either patients seen by rheumatologists (6-8) or community-based samples of adults with self-reported arthritis (9-12). The rheumatology clinic studies have used clinic-confirmed diagnoses but excluded adults treated by primary care physicians and have had small samples (n = 135–232). The community-based surveys have included adults treated by any type of physician or none at all and have had larger samples (n = 122–1424), but these studies have relied on self-report to determine the presence of arthritis. Many such participants did not know what type of arthritis they had (9,10).

A second gap in the literature is the underrepresentation of Hispanic adults in arthritis-specific CAM-use studies in the United States. Two community-based studies conducted 10 or more years ago found that Hispanic and African American adults with arthritis were more likely to use prayer, take herbs orally, or use ointments and were less likely to see a physician for arthritis than were non-Hispanic whites (13,14). Hispanic adults are better represented in studies of CAM use among the general population (4). In studies of CAM use for any purpose among adults in the general population, Hispanics or Latinos have been as likely as non-Hispanic whites to use self-care types of CAM but less likely to have seen a CAM provider (4,5,15-19).

A third shortcoming is that there seems to be little information describing use of specific CAM therapies by type of arthritis, even though the three most common types of arthritis — osteoarthritis, rheumatoid arthritis, and fibromyalgia — differ greatly in disease processes, clinical presentations, persons affected, and the CAM therapies shown to be effective (20,21). Osteoarthritis affects both women and men primarily over age 45, whereas rheumatoid arthritis and fibromyalgia can start at younger ages and affect more women than men (20).

The purpose of this study is to assess the frequency and types of CAM therapy used by Hispanic and non-Hispanic white adults treated by primary care physicians in university-affiliated clinics in Albuquerque, NM, as part of the management of osteoarthritis, rheumatoid arthritis, or fibromyalgia.

## Methods

### Recruitment

Hispanic and non-Hispanic white women and men aged 18–84 years seen in any of six primary care clinics at the University of New Mexico in Albuquerque between June 2000 and May 2001 with a diagnostic code for osteoarthritis, rheumatoid arthritis, or fibromyalgia were eligible for inclusion in the sample. The sampling method is discussed below (Statistical analysis). Of the 1684 sampled patients, 22 (1.3%) had died. For 97 (5.8%), permission to contact was denied by the primary care physician, and for 69 (4.1%), no responding physician could be found. Introductory letters were sent to the 1496 patients (88.8%) for whom physician consent was obtained. Trained bilingual interviewers made telephone calls in English or Spanish to these patients to invite them to participate in the study. Because of the known inaccuracy of ethnicity coding in the clinics, interviewers screened patients during the invitational calls to ensure they self-identified as Hispanic or non-Hispanic white and spoke English or Spanish. During recruitment, we learned that the diagnostic code (ICD-9-CM) for fibromyalgia was not specific to fibromyalgia, but also included people with other muscular conditions. Consequently, some patients denied having fibromyalgia or arthritis when contacted and were excluded.

### Interviews

In-person interviews were conducted by appointment in participants' homes or in a clinic, according to participant preference. The interviewers were university staff trained to conduct interviews for a variety of epidemiological studies.

We used validated and reliable measures from the Centers for Disease Control and Prevention's Behavioral Risk Factor Surveillance System for demographic questions, perceived health status, and comorbidity (22). The 20-item Stanford Health Assessment Questionnaire (HAQ), commonly used in arthritis research, was the measure of functional ability (23). The five-item short form of the Arthritis Helplessness Index, validated by DeVellis and Callahan (24), measured perceived helplessness (25). Fiscella's four-item Medical Skepticism scale (26) assessed beliefs about desire to control one's own health care. We used the Faces Pain Scale to assess average pain over the past week, with a range from 0 = no pain to 10 = worst possible pain (27,28). We used a visual analog scale to evaluate average fatigue over the past week, with a range from 0 = no fatigue to 10 = extreme fatigue (29). A four-item scale measured sleep problems in the past month (30). We also included Marin's five-item Hispanic acculturation scale (31).

We selected CAM items based on a review of the literature and feedback from 39 volunteers in four focus groups. CAM items were included in the survey if they were reported in the CAM literature as commonly used for osteoarthritis, rheumatoid arthritis, or fibromyalgia, or if focus group participants said they were locally used for arthritis. (The Appendix provides a full list of CAM therapies included in the survey.) Participants were also asked to describe other therapies they were using. Current use was defined as use at the time of the interview.

Pilot testing of the questionnaire was conducted in two waves. The first wave involved adults who had arthritis or fibromyalgia and were known to the researchers. In the second wave, interviewers conducted pilot interviews using the modified survey with 18 volunteers recruited from the community who met eligibility criteria for the study. Final revisions were then made to the English-language questionnaire. Previously validated Spanish translations were used for the functional ability HAQ (32); demographic, health status, and comorbidity items (22); Arthritis Helplessness Index (33); and acculturation scale (31). A native Spanish speaker born in Mexico who works in health care in New Mexico translated the remaining measures. Several Spanish-speaking researchers reviewed the translations to ensure a match to the English meaning, proper grammar, and correct spelling and then pilot tested the Spanish version with Spanish speakers who had arthritis.

### Statistical analysis

Participants were randomly selected from the clinic population within strata defined by ethnicity, sex, and diagnostic group. To obtain more rheumatoid arthritis participants, more Hispanics, and more men, we sampled patients in these categories at higher rates than non-Hispanic white women with diagnoses of osteoarthritis or fibromyalgia. The strata were based on the clinic-assigned ethnicity and diagnostic group, although self-reported ethnicity and self-reported diagnosis of fibromyalgia were used in the analysis. Sampling fractions were computed as the ratio of the number of participants who completed an interview to the total number of clinic patients within each stratum. The inverses of the sampling fractions were used as weights in all analyses. SUDAAN software release 8.0.2 (Research Triangle Institute, Research Triangle Park, NC) was used to compute all weighted estimates of proportions and tests of significance by means of a stratified sampling with replacement design (34).

We conducted analyses separately by diagnostic group because the three types of rheumatological conditions are different disease processes with different clinical presentations and patient demographics. For arthritis type, we coded each participant as having primarily rheumatoid arthritis, fibromyalgia, or osteoarthritis. The clinic diagnostic codes were used to identify respondents as having rheumatoid arthritis or osteoarthritis. Because of inaccuracies in the diagnostic coding for fibromyalgia, we used participants' self-reports to code that condition. Participants having more than one type of arthritis were classified in the following order of priority: 1) rheumatoid arthritis, 2) fibromyalgia, and 3) osteoarthritis. Ninety-five patients were classified as having rheumatoid arthritis, 95 as having fibromyalgia, and 422 as having osteoarthritis. One patient with rheumatoid arthritis also had a clinical diagnosis of osteoarthritis, and six reported having fibromyalgia. Nineteen with self-reported fibromyalgia also had a clinical diagnosis of osteoarthritis. Subjects who reported both Hispanic and non-Hispanic ethnicity were coded as Hispanic.

Several survey items were excluded from frequency counts and analyses of CAM use. Multiple vitamins, prayer, and drawing upon religious beliefs were excluded because participants had difficulty distinguishing between use for general health and use specifically for arthritis. Calcium, vitamin D, and folic acid were excluded because participants reported that their physicians had recommended their use for other health reasons.

## Results

### Demographics

A total of 612 primary care clinic patients completed the surveys. Of the 1496 primary clinic patients sent introductory letters, 286 (19.1%) were ineligible per the telephone screening (self-reported ethnicity other than Hispanic or non-Hispanic white or denied having arthritis). Of the 1210 eligible patients, 110 (9.1%) were too ill to participate because of other illnesses, 36 (3.0%) had moved out of the area, 302 (25.0%) refused, 96 (7.9%) had no current contact information, and 54 (4.5%) with valid telephone numbers could not be reached despite repeated calls at different times of the day. The final response rate of completed interviews among eligible patients was 50.6% (612 of the 1210 eligible patients). Information was not available on nonrespondents to compare their characteristics with those of respondents.

Weighted estimates of the proportions of the clinic population with each of several characteristics, both overall and by diagnostic group, are shown in Table 1. An estimated 44.6% were of Hispanic ethnicity and 71.4% were female. About one fourth (26.9%) were aged younger than 55 years, 35.4% were aged 55–64, and 37.7% were aged 65–84. Of the Hispanics, 71.4% self-identified as Spanish American, 20.3% as Mexican American or Mexican, 2.9% as Central or Latin American, and 2.0% as "other" (3.4% were unidentifiable either because the respondents did not know or refused to answer) (data not shown). As expected, clinic patients with rheumatoid arthritis and fibromyalgia were younger and included a higher proportion of women and non-Hispanic whites, and they had more education than the group with osteoarthritis. Patients with fibromyalgia had higher levels of pain in the past week and more frequent physician visits for their rheumatologic condition than did patients with rheumatoid arthritis or osteoarthritis (*P* < 0.01).

### Overall CAM use

Most of the clinic population (90.2%) had ever tried CAM therapies for their arthritis, and 69.2% currently used one or more CAM therapies at the time of the interview (Table 2). CAM users with fibromyalgia had ever tried an average of 5.5 CAM therapies versus 4.4 among those with rheumatoid arthritis and 3.1 among those with osteoarthritis (*P* < .05) (data not shown). CAM users with fibromyalgia currently used an average of 3.9 CAM therapies compared with 2.4 among those with rheumatoid arthritis and 2.1 among those with osteoarthritis (*P* < .05) (data not shown).

### Types of therapies used

Fibromyalgia patients were most likely to use each type of CAM compared to those with rheumatoid arthritis or osteoarthritis (Table 2). Specific CAM therapies currently used by at least 3% of the clinic population in at least one of the diagnostic groups are shown in Table 3. Patients with osteoarthritis most commonly used the nutritional supplements glucosamine (25.2%) and chondroitin (17.9%) and the mind-body therapies of meditation (10.1%) and relaxation techniques (10.0%) to manage their arthritis. Rheumatoid arthritis patients most commonly used relaxation (16.3%), glucosamine (15.8%), meditation (11.1%), and vitamin C (10.0%). Only 6.1% of rheumatoid arthritis patients currently used fish oil supplements, and only 1.3% used supplements containing gamma linolenic acid (GLA) (borage oil, evening primrose oil, and black currant oil). Fibromyalgia patients most commonly used breathing techniques (36.7%), relaxation (28.9%), meditation (27.6%), music therapy (22.8%), glucosamine (20.7%), visualization (19.6%), acupressure (19.5%), massage therapy (17.1%), magnesium (14.1%), and yoga (13.2%). Fibromyalgia patients with a concurrent clinical diagnosis of osteoarthritis were more likely to use glucosamine than were those without osteoarthritis (glucosamine: 49.3% vs 13.8%, *P* = .01; chondroitin: 25.6% vs 6.9%, *P* = .08) (data not shown).

Patients currently used more than 40 different herbs taken orally and more than 30 topical herbal therapies for arthritis. As Table 3 shows, massage therapists, chiropractors, and acupuncturists were the most commonly seen CAM therapists. Within the items worn category, only magnets and copper bracelets were currently used by more than 1% of patients. Respondents were asked about several CAM movement therapies, and they most often named yoga, tai chi, or the Feldenkrais method of movement reeducation. Current use of special diets included an "arthritis diet" high in fish and fresh fruits and vegetables and low in potatoes, tomatoes, eggplant, and peppers. Patients also used several other special diets. Energy therapies included acupressure, reiki, reflexology, therapeutic touch, aromatherapy, and other therapies intended to affect theorized energy fields within and surrounding the body.

### Characteristics of CAM users

Among osteoarthritis and fibromyalgia patients, current use of any type of CAM did not differ by demographic characteristics (Table 4). Among rheumatoid arthritis participants, however, current CAM use was associated with disease duration of 0–5 years and having some college education (*P* < 0.05). Tests for interactions showed the association between ethnicity and current use of any type of CAM differed by diagnostic group. Similar proportions of Hispanic and non-Hispanic white osteoarthritis patients currently used any type of CAM, whereas significantly more non-Hispanic whites with rheumatoid arthritis currently used CAM than did Hispanics with rheumatoid arthritis. We tested other measures of disease burden not shown in Table 4 (including functional ability, fatigue, sleep problems, perceived general health status, and presence of comorbidity) for possible associations with current CAM use, but we found none that was significant. Overall, current use of any type of CAM was significantly higher among women (*P* = .03), patients under age 55 (*P* = .02), and those with some college education (*P* = .003).

### Other aspects of CAM use

Additional results not presented in the tables are described below. The percentages of the clinic population that found CAM therapies somewhat helpful or helped a lot, among those who had ever used that type of CAM, are as follows: mind-body therapies (90.4%); CAM movement therapies (82.7%); CAM therapists (79.8%); energy therapies (79.4%); herbal topical rubs (77.1%); special diets (64.9%); vitamins and minerals (63.0%); herbs taken orally (61.5%); nutritional supplements (57.0%); homeopathic remedies (49.6%); and items worn (36.9%). Gaps between percentages of ever use and current use were greatest for those CAM therapies that fewer patients found useful, such as wearing copper jewelry or magnets, or for costly therapies, such as seeing CAM therapists or using glucosamine and chondroitin. Among those who had ever used CAM, 13.6% of osteoarthritis patients, 17.3% of those with rheumatoid arthritis, and 30.6% of those with fibromyalgia reported that CAM use changed their use of conventional therapies. The most common change reported in open-ended responses was the use of smaller amounts or doses of prescription or over-the-counter medication (52.8%). Of those who had ever tried CAM, 6.6% used only CAM therapies and no conventional treatments. Overall, 22.6% of those who had ever used CAM had never mentioned their CAM use to their medical doctor, 66.6% had told their doctor, 8.0% said their doctor had suggested the therapies, and 2.7% were unsure. More CAM users with fibromyalgia (82.8%) told their doctor about their CAM use than did those with rheumatoid arthritis (69.7%) or osteoarthritis (62.0%) (*P* < .001).

Open-ended responses on reasons for using CAM were in the same rank order by how often they were mentioned in each diagnostic group. Overall, reasons mentioned included the following: to relieve pain (36.1%); to prevent disease progression (14.3%); to feel better (13.7%); to try CAM to see if it would help (13.5%); and because CAM therapies had helped them (9.2%). Sources of information about CAM were also similar across the diagnostic groups. Overall, these sources included family or friends (66.1%), medical doctors (56.1%), magazines or books (34.6%), and radio or television or newspapers (22.6%); only 11.3% named the Internet as a source. Patients who had ever used any type of CAM currently spent from $0 to more than $500 per month on CAM therapies, with 67.7% spending $50 or less per month and 15.1% spending more than $100 per month. Thirty percent of CAM users with fibromyalgia spent more than $100 per month on CAM versus 24.0% of those with rheumatoid arthritis and 9.7% of those with osteoarthritis.

## Discussion

In this study, 90.2% of Hispanic and non-Hispanic white arthritis patients treated by primary care physicians had ever tried CAM for their arthritis, and 69.2% currently used one or more CAM therapies. Overall, 17.2% changed their use of conventional treatments after starting to use CAM, and more than one fifth (22.6%) had never discussed their CAM use with their medical doctor. These findings are important because they indicate that conventional therapies alone are not meeting the needs of arthritis patients. Furthermore, many patients are supplementing or decreasing their medical treatment with or without their physician's knowledge. Because some CAM therapies may interact with conventional medications and treatments, it is important for health care providers to be aware that many arthritis patients are using therapies on their own and to inquire specifically about their patients' CAM use.

The prevalence of CAM use found in this study is higher than the range of 33%–66% reported in previous arthritis studies (6-12). Differing definitions of CAM may partly explain the disparity, as the present study included a broader array of mind-body therapies, energy therapies, and CAM movement therapies than most previous studies. Geographic location may also partly explain the difference, given that national studies of CAM use among the general population have found higher use in the West than in other regions of the United States (5,18). Also, some arthritis studies have included only older adults (9-12) who in previous studies were less likely to use CAM than adults under age 55 (4,5).

As in previous studies, most CAM use in the present study was self-care, with a smaller percentage of people seeing a CAM therapist (4-6,10,12,15). Consistent with some previous studies, current use of any type of CAM in this study was associated with being female, being under age 55, and having some college education, and use did not differ by Hispanic ethnicity (4-6,16,19). National studies on CAM use among the general population found higher income was associated with CAM use (4,5), but the present study and some other arthritis CAM studies found no association between CAM use and income (6,9,11).

The finding that patients with osteoarthritis used a lower average number of CAM therapies than those with fibromyalgia and rheumatoid arthritis conflicts with a previous rheumatology clinic study in which patients with rheumatoid arthritis were less likely to use CAM than patients with osteoarthritis or fibromyalgia (6). Both that study and ours, however, had small numbers of patients with fibromyalgia and rheumatoid arthritis, and the previous study had very few patients with osteoarthritis. Thus, caution should be used in interpreting this finding. In previous studies among adults with arthritis, CAM use was associated with poor perceived general health (10,11), sleep disruption (12), or severe pain (35). In one study, use of three or more types of CAM was associated with longer disease duration (35). In the present study, however, shorter duration of arthritis among respondents with rheumatoid arthritis was associated with current CAM use, and arthritis symptoms were not significantly associated with CAM use. Although similar percentages of Hispanic and non-Hispanic white respondents with osteoarthritis used any type of CAM, use of specific CAM therapies may vary by Hispanic ethnicity. Exploration of this issue is beyond the scope of the present overview.

An important finding of this study is that patterns of CAM use did not necessarily correspond to CAM therapies with evidence of effectiveness for particular types of arthritis. Most striking is the high percentage of patients in each diagnostic group with past and current use of glucosamine and chondroitin. Rigorous scientific studies have shown consistent moderate short-term benefits and safety of glucosamine and chondroitin for osteoarthritis of the knee, but there is no evidence for their effectiveness in rheumatoid arthritis or fibromyalgia (21,36,37). We checked for concurrent osteoarthritis as a possible explanation of use by those with rheumatoid arthritis and fibromyalgia, but we found that none of the participants with rheumatoid arthritis and only half of those with fibromyalgia using glucosamine and chondroitin had osteoarthritis. Another example is the low use of fish oil and GLA-containing supplements among patients with rheumatoid arthritis, even though they have been shown to reduce pain and improve function in those who have rheumatoid arthritis (21,38). Relying on friends, family members, and magazines for information about CAM may have affected participants' access to credible information specific to arthritis type. High use of mind-body therapies was found in the present study within each diagnostic group, especially among those with fibromyalgia. The few efficacy studies of mind-body therapies among patients with fibromyalgia have had equivocal findings, but results are promising for patients with osteoarthritis and rheumatoid arthritis (39).

This study has several limitations. Sample sizes were small for those with rheumatoid arthritis (n = 95) and fibromyalgia (n = 95), although the numbers of osteoarthritis participants (n = 422) and total participants (n = 612) were larger than in previous studies with clinic samples. Results are not generalizable beyond the particular clinic population for several reasons: the response rate was low; men, Hispanics, and those with rheumatoid arthritis were oversampled; the SUDAAN weighted analysis method provided estimates only within the particular clinic population in the study and not a larger population; the low-income, central New Mexican, university-affiliated clinic population is unlikely to be representative of other clinic populations in other locations; and New Mexican Hispanics who self-identify as Spanish Americans may not be representative of diverse Hispanic populations living elsewhere. We used the SUDAAN weighted analysis method to address oversampling certain groups, but this method is intended for use with larger samples (34). Use of CAM ever to manage longstanding arthritis is prone to recall bias, and so we defined current use as use at the time of the interview. There is still the limitation of asking about CAM use at only one point in time. Definitions of CAM are increasingly blurred as some therapies such as vitamins have become part of conventional clinical practice and others such as capsaicin cream are now recommended by the American College of Rheumatology because of evidence of efficacy (3).

Efforts are needed to investigate how best to translate and disseminate CAM efficacy research findings so people will know which CAM therapies may benefit their type of arthritis. Additional research is needed on efficacy and safety of CAM therapies used by arthritis patients, including research on potential negative interactions between CAM therapies and conventional treatments such as medications.

In conclusion, this study found that CAM use was high, potentially costly, and not always communicated to the treating physician among the osteoarthritis, rheumatoid arthritis, and fibromyalgia patients treated by primary care physicians in these clinics. Overall, 69% of the clinic population currently used any type of CAM, and current use was associated with being female, being under 55 years of age, and having a college education. Two thirds of CAM users spent less than $50 per month on CAM, but overall 15%, including 30% of the CAM users with fibromyalgia, spent more than $100 per month on CAM. Two thirds of CAM users in the study discussed CAM use with their medical doctor; the main reason cited for not disclosing CAM use was that the physician did not ask. Physicians and other health care providers should be aware of this high degree of CAM use among arthritis patients and incorporate questions about such use into their routine assessments and treatment planning.

## Figures and Tables

**Table 1:** Patient Characteristics by Diagnosis, Percentages Weighted by Inverse of Sampling Fraction, Survey on Use of Complementary and Alternative Medicine (n = 612), New Mexico, 2001–2002

**Characteristics**	**Diagnoses**				

	**Osteoarthritis** **(n=422)**	**Rheumatoid** **Arthritis** **(n=95)**	**Fibromyalgia** **(n=95)**	**All** **(n=612)**	**Pearson** **Chi-square** ***P* Value**

**Ethnic Group**					

Hispanic (%)	47.8	49.2	30.2	44.6	.001
Non-Hispanic white (%)	52.2	50.8	69.8	55.4	

**Sex**					

Female (%)	66.6	84.6	85.6	71.4	<.001
Male (%)	33.4	15.4	14.4	28.6	

**Age at interview (years)**					

18-54 (%)	21.5	41.2	43.2	26.9	<.001
55-64 (%)	34.9	31.1	39.2	35.4	
65-74 (%)	27.9	22.9	16.9	25.5	
75-84 (%)	15.7	4.8	0.8	12.2	
Mean	62.6	56.0	54.8	60.6	<.001
Median	61.8	57.8	55.2	59.9	

**Education**					

Did not graduate from high school (%)	28.9	21.0	7.7	24.3	<.001
High school graduate or GED (%)	19.5	26.6	22.0	20.5	
Some college or technical school (%)	21.9	27.1	39.8	25.7	
College graduate (%)	29.5	25.3	30.5	29.4	
Unknown (%)	0.2	0.0	0.0	0.1	

**Annual household income**					

Less than $25,000 (%)	64.4	66.2	66.9	65.0	.95
$25,000 to $50,000 (%)	18.2	15.0	16.8	17.7	
$50,000 or more (%)	12.8	13.9	14.0	13.1	
Unknown (%)	4.6	4.9	2.3	4.2	

**Frequency of visits to medical doctor for arthritis (times per year) **					

Never or <1 (%)	26.6	10.5	14.3	23.2	<.001
1-2 (%)	24.9	30.2	17.4	23.9	
3-5 (%)	30.8	32.9	30.0	30.8	
>5 (%)	16.0	26.4	38.3	20.9	
Unknown (%)	1.6	0.0	0.0	1.2	

**Disease duration (years)**					

0-5 (%)	34.0	21.0	32.4	32,8	.07
6-14 (%)	33.1	41.7	36.8	34.4	
15+ (%)	32.8	37.2	30.3	32.6	
Unknown (%)	0.2	0.0	0.6	0.2	
Mean	12.6	15.3	12.0	12.6	.12
Median	9.1	11.8	8.2	9.1	

**Pain in last week (0-10 visual scale)**					

Low (0-4) (%)	38.1	41.2	23.2	35.5	.004
Moderate (5-7) (%)	38.9	33.8	36.1	38.0	
High (8-10) (%)	21.6	25.0	40.7	25.5	
Unknown (%)	1.3	0.0	0.0	1.0	
Mean	5.3	5.3	6.6	5.6	<.001
Median	5.0	5.0	6.3	5.2	

**Table 2:** Weighted Estimates of Ever and Current Use of Complementary and Alternative Medicine Type, by Diagnosis, Survey Results (n = 612), New Mexico, 2001–2002

**Types of CAM**	**Diagnoses**				

	**Osteoarthritis** ** (n=422)** **%**	**Rheumatoid** **Arthritis** ** (n=95)** **%**	**Fibromyalgia** **(n=95)** **%**	**All** **(n=612)** **%**	**Pearson** **Chi-square** ***P* Value**

**Any type of CAM**					

Ever	89.4	86.4	94.8	90.2	.08
Current	66.7	70.7	78.3	69.2	.06

**Nutritional supplements**					

Ever	55.4	59.8	72.8	59.0	.007
Current	34.1	26.6	36.5	34.1	.21

**Vitamins and minerals**					

Ever	16.8	28.8	42.7	22.6	<.001
Current	12.1	20.8	32.9	16.6	<.001

**Herbs taken orally**					

Ever	23.5	30.2	50.8	29.1	<.001
Current	10.1	15.6	26.7	13.6	.002

**topical herbal rubs**					

Ever	46.2	48.3	64.9	49.9	.005
Current	23.1	15.6	36.5	25.1	.004

**Items worn**					

Ever	28.2	43.9	39.3	31.3	.006
Current	8.3	10.2	12.0	9.2	.56

**Mind-body therapies**					

Ever	27.9	42.5	59.4	34.8	<.001
Current	23.7	31.1	48.9	29.0	<.001

**Energy therapies^a^**					

Ever	18.3	28.1	55.0	25.9	<.001
Current	7.6	13.2	34.3	13.0	<.001

**Movement therapies^b^**					

Ever	15.1	14.8	32.6	18.4	.005
Current	8.4	5.8	20.7	10.6	.01

**CAM therapists**					

Ever	32.2	45.0	64.6	39.2	<.001
Current	7.6	13.7	32.2	12.7	<.001

**Dietary approaches**					

Ever	10.1	27.4	29.4	14.9	<.001
Current	6.4	17.4	21.8	10.1	<.001

**Homeopathy**					

Ever	3.3	7.1	9.3	4.8	.10
Current	0.9	0.0	3.6	1.3	.06

a Therapies such as acupressure, reflexology, reiki, therapeutic touch, and aromatherapy.

b Primarily yoga, tai chi, and Feldenkrais.

**Table 3:** Weighted Estimates of the Percentages of Osteoarthritis, Rheumatoid Arthritis, and Fibromyalgia Patients Using Complementary and Alternative Medicine at Time of Interview, Survey Results (n = 612), New Mexico, 2001–2002^a^

**Type of Complementary and Alternative Medicine**		**Diagnoses**				

		**Osteoarthritis** **(n=422)** **% **	**Rheumatoid** **Arthritis** **(n=95)** **% **	**Fibromyalgia** **(n=95)** **% **	**All** **(n=612)** **% **	**Pearson** **Chi-square** ***P* Value **
**Nutritional supplements**	**Glucosamine**	25.2	15.8	20.7	23.7	.09
	**Chondroitin**	17.9	7.5	10.6	15.8	.006
	**MSM**	4.6	4.2	9.9	5.6	.26
	**Flaxseed oil**	1.9	5.3	9.9	3.6	.02
	**Vinegar**	4.5	5.1	3.4	4.3	.83
	**Fish oil**	3.0	6.1	6.4	3.9	.27
	**Cod liver oil**	1.9	0.9	3.6	2.2	.35
	**GLA (Gamma linolenic acid) (e.g., borage oil, black currant oil, evening primrose oil)**	0.6	1.3	3.5	1.2	.31
**Vitamins and minerals**	**Magnesium**	3.1	4.2	14.1	5.3	.02
	**Vitamin E**	4.3	8.6	11.1	5.9	.08
	**Vitamin C**	4.1	10.0	9.7	5.6	.06
	**Vitamin B12**	3.4	4.7	5.0	3.8	.74
	**Niacin**	0.2	0.0	5.5	1.2	.06
	**Zinc**	1.3	3.1	3.9	1.9	.32
	**Vitamin B Complex**	0.9	2.4	4.0	1.2	.23
	**Selenium**	0.3	0.0	3.7	0.9	.15
**Herbs taken orally**	**Garlic**	4.4	6.9	8.2	5.3	.34
	**Ginger**	1.4	4.6	4.0	2.1	.16
	**Valerian root**	1.1	2.9	4.6	1.9	.19
	**Aloe vera**	1.0	3.4	3.1	1.6	.29
	**St. John’s wort**	0.0	1.7	3.3	0.7	.09
	**Turmeric**	0.5	1.7	3.4	1.1	.24
**Homeopathy**	**Arnica (oral)**	0.3	0.0	3.6	0.9	.15
**topical rubs**	**Tiger balm**	5.1	4.2	10.8	6.1	.19
	**Arnica cream**	2.2	1.7	5.1	2.7	.42
	**Chamomile oil**	1.0	0.0	4.9	1.6	.02
	**Capsaicin cream**	4.6	0.9	3.6	4.1	.03
	**Sesame oil**	0.3	0.0	3.6	0.9	.09
	**Horse liniment**	1.3	3.9	2.8	1.8	.33
	**Traumeel ointment**	1.2	1.7	4.6	1.8	.33
	**Rosemary oil**	0.5	1.2	3.0	1.0	.34
	**Aloe vera cream**	1.1	0.0	3.1	1.4	.02
**Items worn**	**Magnets**	4.6	7.6	9.6	5.7	.22
	**Copper jewelry**	4.2	3.8	2.8	3.9	.79
**Mind-body therapies**	**Relaxation techniques**	10.0	16.3	28.9	14.0	.001
	**Meditation**	10.1	11.1	27.6	13.5	.004
	**Breathing techniques**	8.4	9.8	36.7	13.8	<.001
	**Music therapy**	6.2	8.8	22.8	9.5	.002
	**Visualization**	6.8	7.4	19.6	9.2	.02
**Energy therapies**	**Acupressure**	2.4	2.9	19.5	5.7	.001
	**Reflexology**	0.7	2.0	9.8	2.5	.02
	**Aromatherapy**	1.1	3.2	7.4	2.4	.05
	**Therapeutic touch**	1.1	2.7	4.8	1.9	.16
	**Self-massage**	0.2	3.2	0.9	0.5	.19
	**Electromagnetic**	1.5	2.0	4.1	2.0	.54
	**Reiki**	0.4	0.9	3.5	1.1	.32
**Movement therapies**	**Yoga**	4.9	4.9	13.2	6.5	.09
	**Tai chi**	2.6	0.9	6.3	3.2	.09
	**Feldenkrais**	0.5	0.0	5.1	1.3	.04
**CAM therapists**	**Massage therapists**	4.8	4.4	17.1	7.1	.01
	**Chiropractors**	1.5	1.7	10.3	3.1	.05
	**Acupuncturists**	2.1	2.9	5.5	2.8	.46
	**Myofascial therapists**	0.2	0.0	3.2	0.8	.14
	**Osteopathic doctor**	0.0	0.9	3.2	0.7	.13
**Dietary approaches**	**“Arthritis diet”**	1.4	8.5	4.4	2.4	.02
	**Fasting or cleansing diet**	0.2	4.0	7.3	1.8	.006
	**Hypoglycemic diet**	0.4	0.0	5.0	1.2	.04
	**Vegetarian diet**	1.2	1.7	3.6	1.7	.45

a Table includes complementary and alternative medicine (CAM) items used by 3% or more of patients in at least one diagnostic group. See Appendix for complete list of CAM items included in the survey.

**Table 4:** Weighted Estimates of Current Use of Any Type of Complementary and Alternative Medicine, by Patient Characteristics and Diagnostic Group, Survey Results (n = 612), New Mexico, 2001–2002

**Patient Characteristics**		**Diagnoses**				

		**Osteoarthritis** **(n=422)** **%**	**Rheumatoid** **Arthritis** **(n=95)** **%**	**Fibromyalgia** **(n=95)** **%**	**All** **Participants** **(n=612)** **%**	**Test of** **Interaction** ***P* Value**
**Ethnic group**	**Hispanic **	65.5	54.8	70.9	65.4	NA^a^
	**Non-Hispanic White**	67.8	86.0	81.5	72.2	NA
	**(Pearson chi-square *P *value)**	(0.62)	(0.001)	(0.27)	(0.08)	.03
**Sex**	**Female**	69.3	71.8	79.6	71.9	NA
	**Male**	61.5	64.0	70.7	62.4	NA
	**(Pearson chi-square *P *value)**	(0.11)	(0.60)	(0.55)	(0.03)	.98
**Age at interview (years)**	**18-54**	71.9	77.9	84.8	76.4	NA
	**55-64**	68.3	70.1	78.1	70.4	NA
	**65-84**	62.8	60.6	62.9	62.7	NA
	**(Pearson chi-square *P *value)**	(0.30)	(0.37)	(0.26)	(0.02)	.84
**Education**	**Did not graduate from high school **	60.7	57.0	49.0	59.8	NA
	**High school graduate or GED **	65.7	59.8	69.1	65.9	NA
	**Some college or technical school**	66.6	72.7	81.2	71.3	NA
	**College graduate **	73.1	91.3	88.7	77.3	NA
	**(Pearson chi-square *P *value)**	(0.24)	(0.02)	(0.14)	(0.009)	.44
**Annual household income**	**Less than $25,000 **	67.0	66.9	75.9	68.7	NA
	**$25,000 to $50,000**	62.2	80.4	82.1	66.8	NA
	**$50,000 or more **	72.0	82.2	90.0	76.3	NA
	**(Pearson chi-square *P *value)**	(0.54)	(0.36)	(0.45)	(0.34)	.68
**Frequency of visits to medical doctor for arthritis (times per year)**	**Never or < 1 **	60.4	82.9	51.3	60.1	NA
	**1-2 **	69.0	84.6	81.3	72.0	NA
	**3-5 **	69.1	59.1	86.1	71.4	NA
	**>5**	67.1	64.3	81.0	71.7	NA
	**(Pearson chi-square *P* value)**	(0.52)	(0.08)	(0.25)	(0.14)	.15
**Duration of disease (years)**	**0-5**	60.3	91.5	74.6	64.3	NA
	**6-14**	67.4	60.3	79.8	69.3	NA
	**15+**	72.9	70.6	80.1	74.0	NA
	**(Pearson chi-square *P* value)**	(0.09)	(0.01)	(0.85)	(0.13)	.11
**Pain in last week**	**Low **	64.3	77.8	85.0	67.9	NA
	**Moderate**	66.5	70.9	81.3	69.4	NA
	**High**	72.9	58.5	71.9	71.6	NA
	**(Pearson chi-square *P* value)**	(0.36)	(0.33)	(0.46)	(0.77)	.23

a NA indicates not applicable.

## Appendix

Complementary and Alternative Medicine Items Included in Survey of Hispanic and Non-Hispanic White Women and Men Aged 18–84 (n = 612), New Mexico, 2001–2002

**Nutritional supplements:** glucosamine, chondroitin, MSM, SAMe, shark cartilage, bovine cartilage, collagen, cod liver oil, fish oil, flaxseed oil, GLA-containing supplements (borage oil, black currant oil, evening primrose oil), vinegar, bromelain, quercetin, noni juice, pregnenolone, DLPA, glutathione, malic acid. **Vitamins & minerals**: Multiple vitamin^a^, calcium^a^, vitamin D^a^, vitamin C, vitamin E, beta carotene, niacin, vitamin B5, vitamin B12, folic acid^a^, magnesium, selenium, boron, copper, zinc. **Oral herbs**: aloe vera, angelica root, black cohosh, boswellia (guggul), burdock root & seed, cactus, calendula, cat's claw, cayenne, celery seed, coix, corydalis, devil's claw, eucalyptus, fennel, feverfew, foti (Hou Shu Wu), garlic, gentian, ginger, kava kava, thundergod vine, licorice, oregano, St. John's wort, stinging nettle, turmeric, valerian root, white willow bark, wild yam, yucca. **Topical oils, rubs and ointments**: arnica cream, calendula, capsaicin creams, chamomile oil, clay, coriander cream, DMSO, horse liniment, linseed oil, MSM creams, pine tree sap, rosemary oil, sesame oil, tiger balm, traumeel or traumed ointment, volcanico. **Items worn**: acupressure beads or seeds, copper jewelry, herbal plasters, infrared wraps, magnets, Q-ray ionically charged bracelet. **Mind-body therapies**: meditation, visualization, special breathing techniques, relaxation techniques, music therapy, draw upon religious or spiritual beliefs^a^, pray^a^, attend religious services regularly^a^. **Energy therapies**: acupressure, aromatherapy, biofeedback, cranio-sacral therapy, hypnotism, polarity, reflexology, reiki, therapeutic touch. **Movement therapies**: yoga, tai chi, qigong, pilates, Feldenkrais method ("Awareness Through Movement"), Alexander movement techniques, Trager approach ("Mentastics"). **CAM therapists**: doctor of oriental medicine or acupuncturist, ayurvedic doctor, chiropractic doctor, curandero/curandera, herbalist, hypnotist, iridologist, massage therapist, myofascial therapist, naturopathic doctor, osteopathic doctor, religious leader, spiritual healer. **Dietary approaches**: "arthritis diet" with fish, fresh fruits, and vegetables except potatoes, tomatoes, eggplant, peppers; ayurvedic diet; fasting and cleansing diets; hypoglycemic diet; vegetarian diet.

a Item excluded from analyses.
